# Human–machine interaction in computational cancer pathology

**DOI:** 10.1016/j.esmorw.2024.100062

**Published:** 2024-08-13

**Authors:** A. Syrnioti, A. Polónia, J. Pinto, C. Eloy

**Affiliations:** 1 Department of Pathology, School of Medicine, Aristotle University of Thessaloniki, Thessaloniki, Greece; 2 Pathology Laboratory, Institute of Molecular Pathology and Immunology of University of Porto (IPATIMUP), Porto; 3 School of Medicine and Biomedical Sciences, Fernando Pessoa University, Porto; 4 Pathology Department, Medical Faculty of University of Porto, Porto, Portugal

**Keywords:** computational pathology, cancer diagnosis, artificial intelligence, synergy

## Abstract

The performance of the augmented pathologist that works in synergy with artificial intelligence (AI) is generally accepted as the most accurate in comparison to AI standing alone and the general pathologist standing alone. Human–machine interactions triggered by the synergic daily work give rise to trust-related concerns and potential biases that need to be addressed. The long-term use of AI requires actions to prevent deskilling of the pathology workforce, and to ensuring appropriate education of future generations. Establishment of clear guidelines for the verification and validation of AI tools is crucial for the maintenance of high-quality cancer pathology.

## Highlights

• Synergic AI pathologist models can enhance accuracy and efficiency in cancer diagnosis.
• Human–machine interactions are tailored by sex, age, place of practice, and experience level of the pathologist using AI.
• Concerns on AI clinical use include implementation issues, potential biases, and deskilling.
• Validation and quality control are essential for ensuring AI reliability.

The digital transformation of pathology laboratories, taken as the progressive automation and standardization of procedures that ultimately aim to produce high-quality whole slide images for diagnosis and respective computational-based analysis (so called, computational pathology), represent the most recent challenge in pathology. Since the early days, the demonstration of the potential of computational pathology, namely of artificial intelligence (AI), to change pathology practice followed twisted paths. Connotated as a technology designed to substitute the pathologist in its multiple tasks, reverberating as a pending threat, was (and still is) repeatedly announced as a salvation from the inherent human limitations of pathologists.^1^ Numerous works and challenges stimulated the confront between the pathologists and the AI-equipped computers on finalizing a very specific task, often demonstrating the superior performance of the machine.^2^, ^3^, ^4^ The natural consequence of this unframed marketing was the immediate scepticism of the general pathology community regarding AI^5^ that was only relieved after the concept of the synergic model of reporting and the recognition that, for now, AI should be regarded as a tool.^4^^,^^6^^,^^7^

The performance of the augmented pathologist that works together, in synergy, with AI as a tool has been demonstrated as the best one, in comparison to AI standing alone and the general pathologist standing alone.^4^^,^^7^, ^8^, ^9^ Similar findings have been reported in the setting of radiology, namely in digital mammography reporting.^10^ If AI can accelerate precise quantifications, alert for small events and/or contribute to homogeneous qualifications such as grading, the human brain is still requested for the integrative exercise.^11^ For the time being, AI algorithms are mostly dedicated to organ-specific and often disease-specific tasks, precluding when working alone, the differential diagnosis activity that characterizes the essence of medicine. Under these premisses, the prevailing opinion of pathologists is that AI will predominantly function as an assisting tool, being those concerns about potential substitution being less and less frequent.^12^, ^13^, ^14^, ^15^, ^16^

Currently, there is an expanding global interest by pathologists on this technology.^16^ In a Delphi study exploring the future role of AI in pathology, experts reached the consensus that AI will be integrated into routine pathology workflows by 2030.^16^ In fact, the majority of pathologists perceive that AI holds the potential to enhance the quality and efficacy of their daily work^12^, ^13^, ^14^, ^15^^,^^17^, ^18^, ^19^ being those who exhibited greater receptiveness to the integration of AI in their daily work compared with psychiatrists, radiologists, and surgical specialists.^15^ Dealing with overloaded routines often due to shortage of manpower, pathologists envision AI fulfilling a broad spectrum of roles within the diagnostic workflow,^17^ including prioritizing samples, pre-ordering additional tests, as well as assisting with time-consuming quantification tasks, such as counting events.^20^ These observations are in line with a study on prostate biopsies that identified significant reduction in time for reporting, second opinion requests, and number of immunohistochemistry tests, as primary advantages of the use of AI tools in pathology.^7^ The results of this study also impact in the quality of cancer reporting.^7^ Obviously, improving quality is also a driver to the adoption of AI solutions that can be used as a second observer to flag potentially overlooked features^7^^,^^20^^,^^21^ or to help organizing information in a synoptic and structured report. In the context of precision pathology for cancer diagnosis, AI has the potential to assist pathologists not only in diagnosis, but also in the quantification of biomarkers, prognosis assessment, and even prediction of underlying molecular alterations.^22^ Examples include the development of AI-based methods for programmed death-ligand 1 (PD-L1) and microsatellite instability (MSI) status prediction.^23^^,^^24^ Others forecast therapeutic outcomes for targeted agents, immune checkpoint inhibitors, and chemotherapeutic medications,^25^^,^^26^ still without clinical application.

The clinical use of AI in the range of pathology, namely supporting the characterization of several cancer models, is tailored by several factors. Some of these factors arise at the individual level, generated by the abovementioned synergic interaction between the machine and the singularities of each one of us. Certain reports suggested that male pathologists tend to exhibit greater optimism and less apprehension regarding the integration of AI into clinical practice, compared with females.^12^^,^^19^ Interestingly, in another study, pathologists aged >40 years demonstrated a greater sense of confidence regarding error reduction with AI tools and anticipated an earlier adoption of AI into practice.^12^ In another study, however, age was not found to significantly influence the general perception of AI.^19^ In addition, the level of experience may also influence both the general perception, and the interactions with AI. In the aforementioned Delphi study, those with less than a decade of experience on the subject more frequently supported the idea that digital pathology, along with the interpretation of computationally-derived assessments, would become routine by 2030, compared with those with longer experience.^16^ In a study involving oral pathologists in India, knowledge regarding AI in pathology was significantly correlated with years of experience (seniors).^18^ A pioneer study reporting on human–machine interaction also revealed that expert pathologists and residents may differ in the extent to which they allow the use of an AI tool to impact their final diagnosis on a series of breast lesions.^4^ In fact, senior pathologists tended to use AI in their benefit in a better way than residents.

The benefits of AI in clinical use may also vary based on the scale and nature of the workload in the pathology department. It has been reported that with the assistance of AI tools, generalists in smaller departments could enhance their consensus with specialists in terms of Gleason grading of prostate biopsies, thereby reducing variability and enhancing accuracy.^8^^,^^20^ Specialized centres, however, in the context of national screening programs, may use AI to prioritize samples, screening out negative cases, aiming to alleviate workload.^20^ In addition, in contrast to community pathologists, academic pathologists have demonstrated more favourable attitudes toward AI tool integration, with a higher likelihood of foreseeing cost-effectiveness and emphasizing quality assurance.^12^

AI adoption in clinical practice has been blocked by implementation issues, particularly due to the lack of mature digital pathology workflows in many institutions worldwide.^11^^,^^12^^,^^18^^,^^20^^,^^27^^,^^28^ Trust-related concerns have also emerged, particularly regarding errors that can arise from the AI tool or human–machine interaction. These include the so-called erroneous errors stemming from mislabelling, and the data limitations such as missing or skewed data manipulated during the algorithm development, introducing biases and compromising generalizability of the results regardless the user.^29^

Another pertinent concern is related with the caducity of the AI algorithms that may provide incorrect classifications/information since their deployment occurred before update or revision of the classifications and staging systems used at a the diagnostic moment.^29^ Another type of error that raises mistrust and justifies surveillance of the functioning of AI tools is related with the concept of the designated unsafe failure mode, where the AI tool gives a result despite insufficient information or training, jeopardizing the reliability of the diagnosis.^30^ Equally critical is the AI’s insensitivity to impact, wherein computational tools consider all types of errors to be equal, failing to discern that various diagnostic errors can yield vastly different prognostic and therapeutic outcomes for patients.^29^

A commonly held belief is that explainability may help bolster trust in AI tools, especially when explanations illustrate the AI making decisions in a manner that users find relatable^21^; however, some advocate that they do not deem it necessary to comprehend every step in an algorithm’s decision-making process, as long as its performance and reproducibility are verifiable.^17^^,^^20^ Others also express the need to understand how an AI tool was validated, particularly in terms of the data utilized for training and testing.^20^^,^^21^ In an online experiment, the participants’ reliance on AI recommendations for diagnosis was found to be associated more with general beliefs in its usefulness rather than the information provided about the algorithm.^31^ Regardless the impact the explainability may have in trust and error reduction during AI clinical use, it is our duty, as a community, to pledge for the maintenance of the concept of medicine based on the evidence, such as periodic statistic data on the algorithm performance as well as information on the patterns of reply, rather than in remote believes.

As stressed before, the result of the human–machine interaction generates consequences beyond the mistrust that are hard to control, to predict, and to avoid since these are effects that may pass by unnoticed. One example is related with the pathologist performance that, after prolonged use of an AI tool with a good performance, starts overly focusing on the AI-highlighted events, potentially overlooking small yet crucial features in the entire tissue section.^20^ It has been argued that relying too heavily on AI is risky, as it may lead to this automation bias, making pathologists more susceptible to diagnostic errors.^27^ This bias effect was demonstrated in a study examining the diagnostic accuracy of pathologists reviewing prostatic biopsy specimens with and without AI assistance. In this study, in a small number of cases, pathologists altered an initially correct interpretation to an incorrect one based on the AI inaccurate result.^32^ Since AI tools are also used to decrease interobserver variability, it is necessary to understand to what extent this bias effect or just a tendency to converge in the opinions, are contributing to homogenize results. Similar effects were already identified including those in the group of the cognitive biases when the pathologists overly rely on the initial diagnosis provided by an AI tool or concentrates to only seek out evidence that confirms the AI diagnosis, potentially overlooking contradicting information.^29^

Long-term consequences of AI use are also under consideration, mainly for those who are exclusively reviewing cases with AI assistance, that might have their skills gradually declining.^33^ If AI triages or handles all simpler or more common cases, pathologists may become less familiar with them over time.^33^ The same holds true regarding the long-term consequences on the newer generations of pathologists that will not learn how to work without AI support. It is widely accepted that the maintenance of a traditional training programme is still necessary to warrant the robustness of the future generations of diagnosticians, who should probably be subspecialized to be able to survey AI peformance.^34^ In the scope are also extreme scenarios where AI manages most straightforward cases, leaving the pathologist to deal with the most challenging ones, thereby elevating the complexity of their work.^20^ Or ultimately, at a stage when AI capabilities are general and pathologists may also perceive a diminished value or need for their expertise, potentially resulting in decreased job satisfaction.^33^ A major concern lies within real ethical and legal challenges, particularly in relation to medico-legal liability for errors resulting from AI-based decisions, namely on those results that cannot be verified (or even used) by the pathologist.^12^^,^^16^^,^^20^^,^^35^

Ultimately, in the age of precision medicine, where AI tools play a significant role not only in cancer detection but also in multimodal biomarker evaluation, as well as guiding therapeutic selection, an interpretable verification of their performance is required to ensure patient safety. The appropriate quality control of AI performance should be monitored by the pathologist based upon solid knowledge on the functioning of the AI algorithm, something that can only be achieved by promoting literacy of the pathologist on the subject.^36^^,^^37^ In addition, external validation across large-scale databases, prospective studies, along with datasets representing diverse ethnic groups and originating from various institutions is crucial to ensure generalizability in terms of both population distribution, and site-specific characteristics, such as variations in cutting thickness, staining techniques, or scanning protocols.^38^ This site-specific variability aggravated by the well-known fragility of the tissue processing leads to variable tissue preparation types for computational analysis.^39^^,^^40^ Is it easy to admit there is a tremendous need for standardization of preanalytical variables and rigorous quality control at each stage of tissue processing to guarantee the generation of high-quality materials required for effective utilization by computational pathology tools.

In conclusion, the adoption of the synergic model of work between the pathologist and computationally-derived tools holds the potential to transform the pathology practice by contributing to enhanced diagnostic accuracy, reduced turnaround time, as well as offering predictive capabilities for therapeutic responses. Nevertheless, implementation issues, trust-related concerns, and potential biases need to be addressed. Additionally, preventing deskilling of the already depleted pathology workforce,^41^ ensuring appropriate education of future generations, along with establishing clear guidelines for the verification and validation of AI tools, firmly highlight medico-legal liability in case of error, and standardization of tissue processing is crucial.
